# Genomic markers on synthetic genomes

**DOI:** 10.1002/elsc.202100030

**Published:** 2021-11-10

**Authors:** Hao‐Qian Zhao, Wen‐Qing Wei, Chao Zhao, Ze‐Xiong Xie

**Affiliations:** ^1^ Frontiers Science Center for Synthetic Biology and Key Laboratory of Systems Bioengineering (Ministry of Education) School of Chemical Engineering and Technology Tianjin University Tianjin P. R. China

**Keywords:** genomic marker, PCRTag, recoding, synthetic genome, watermark

## Abstract

Genome synthesis endows scientists the ability of *de novo* creating genomes absent in nature, by thorough redesigning DNA sequences and introducing numerous custom features. However, the genome synthesis is a labor‐ and time‐consuming work, and thus it is a challenge to verify and quantify the synthetic genome rapidly and precisely. Thus, specific DNA sequences different from native genomic sequences are designed into synthetic genomes during synthesis, namely genomic markers. Genomic markers can be easily detected by PCR reaction, whole‐genome sequencing (WGS) and a variety of methods to identify the synthetic genome from native one. Here, we review types and applications of genomic markers utilized in synthetic genomes, with the hope of providing a guidance for future works.

**Abbreviations**
ORF: open reading frame
PCR: polymerase chain reaction
WGS: whole‐genome sequencing

## 1 INTRODUCTION

The development of DNA synthesis and assembly technologies advances the whole genome synthesis [1, 2]. Synthetic genomes are designed from natural genomic sequences with new features, including minimized genomes, codon‐reduced genomes and evolution‐inducible genomes, etc. [3]. Then, the synthetic genome is chemically assembled from scratch and transplanted to recipient cells to replace the native genome [4, 5]. However, it is challenging to verify the replacement of a native genome by corresponding synthetic one, due to the synthesis is a laborious work. Therefore, genomic markers are introduced into synthetic genomes to address the challenge and to differentiate the synthetic genome and the native one. Genomic markers are specific DNA sequences that differ from native genomes. They are landmarks of synthetic genomic sequences, and can be used to verify and quantify the synthetic content of genome or isolate individuals with specific genotypes from populations.

## 2 TYPES OF GENOMIC MARKERS

**FIGURE 1:**
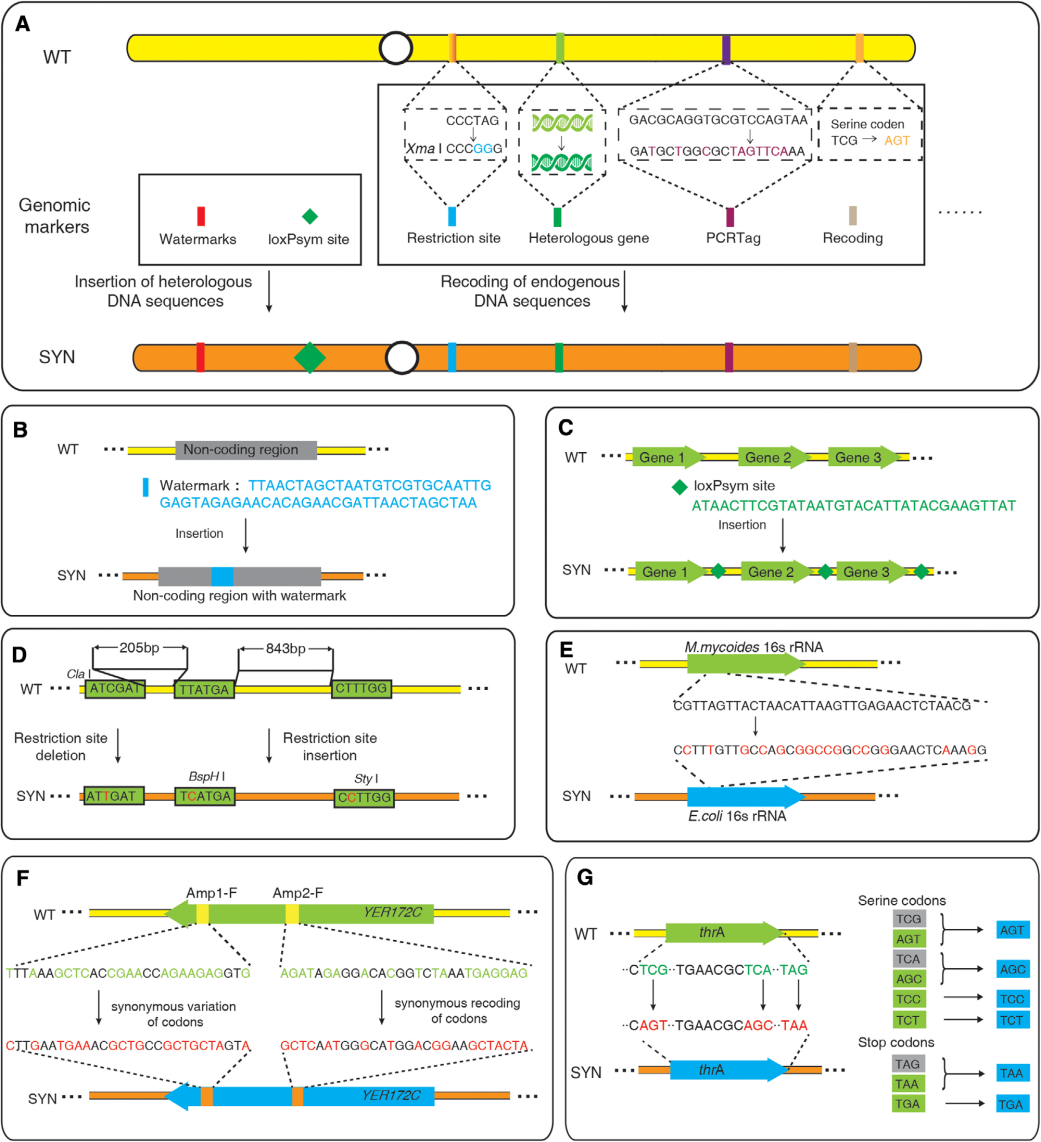
Types of genomic markers on synthetic genomes. (A) Genomic markers are classified into two types, insertion of heterologous DNA sequences and recoding of endogenous DNA sequences. (B) Watermarks. Heterologous DNA sequences are inserted into non‐coding regions of JCVI‐syn1.0 to work as watermarks. (C) Recombination sites. LoxPsym sites are inserted into 3′UTR of nonessential genes on synthetic yeast chromosomes. (D) Restriction enzyme sites. Restriction enzyme sites are introduced or removed from the wild‐type yeast chromosome V (wtV) by synonymous codon recoding. (E) Heterologous gene. In the JVCI‐syn3.0 genome synthesis, the 16S rRNA gene was replaced with a phylogenetically distant *E. coli* counterpart. (F) PCRTag. PCRTags are synonymous recoded short sequences on synthetic yeast chromosomes. (G) Recoding. In the synthetic *E. coli* genome, serine codons TCG and TCA are genome‐widely replaced by synonymous codons AGT and AGC, respectively. Similarly, the stop codon TAG is recoded to TAA

Various genomic markers are incorporated into different synthetic genomes. Basically, genomic markers can be classified into two types: insertion of heterologous DNA sequences and recoding of endogenous DNA sequences (Figure 1A). Inserted genomic markers include watermarks [5, 6] and recombination sites [7, 8, 9, 10, 11, 12, 13].

Watermarks are heterologous DNA sequences that encode unique identifiers but not translate into peptides [14]. For example, four watermarks of about 1‐kb in length were inserted into the synthetic *Mycoplasma mycoides* genome JCVI‐syn1.0 at places where the insertion of additional sequence was demonstrated not to interfere with cell viability [5]. To encode unique sequences of watermarks, information including the names of 46 authors, the website address of institute, and the quotation of “to live, to err, to fall, to triumph, to recreate life out of life” was translated into abbreviations of amino acid, which were further translated to corresponding DNA codons [15]. PCR reaction and WGS methods were further used to identify the synthetic genome from native one by tracking watermarks. Meanwhile, restriction enzyme sites of *Asc*I and *Bss*HII were designed into every watermark. The identification of synthetic genome was also performed by enzyme digestion and pulsed‐field gel electrophoresis (PFGE) [5] (Figure 1B).

In synthetic chromosomes of *Saccharomyces cerevisiae*, loxPsym sites are inserted into 3′ UTR of nonessential genes. The inserted loxPsym sites make it possible to facilitate inducible recombination events that lead to site‐specific rearrangements in genome scale (namely SCRaMbLE), generating diverse genotypes and evolutional genomes [16, 17, 18, 19, 20, 21, 22, 23, 24, 25, 26, 27] (Figure 1C). To characterize rearranged genomes, loxPsym sites are also worked as landmarks to identify novel structural junctions. For example, the ring synthetic yeast chromosome V (ring_synV) was divided into 170 segments by 170 inserted loxPsym [26]. DNA segments between two loxPsym sites were numbered from left to right (from 1 to 170). Therefore, the two segments flanking a loxPsym site define a junction. WGS was performed to characterize the rearranged ring_synV. Unmapped reads carrying a loxPsym site was first trisected to a loxPsym site and its two flanking extremities. Each of the latter was mapped to the reference genome to identify novel junctions [26, 28]. Aside from the 170 original loxPsym junctions in ring_synV, 53 novel structural junctions were figured out, indicating that complex neochromosomes were generated during the continuous SCRaMbLE.

Recoded genomic markers are generated by means of synonymous altering native DNA sequences. These genomic markers include specific restriction enzyme sites, short recoded sequences, and recoded sense codons with synonymous substitutions in open reading frames (ORFs). In the yeast genome, intergenic and intronic sequences house uncharacterized regulatory sequences, which could be disrupted by base changes. Therefore, restriction enzyme sites are introduced or removed within ORFs by base substitution. For example, in the synthetic yeast chromosome V (synV), a *Bsp*HI restriction site “TCATGA” was introduced to *YER013W* gene by synonymously recoding “TTATGA”[9] (Figure 1D). Short recoded sequences are generated by synonymous nucleotide alterations within ORFs [29]. The alterations make it possible to generate different DNA sequences without affecting the function of genetic element. For example, the 16S rRNA gene of synthetic *M. mycoides* genome JCVI‐syn3.0 was replaced with a phylogenetically distant *Escherichia coli* counterpart, which could be used to distinguish the minimized genome JCVI‐syn3.0 from its parent JCVI‐syn1.0 by PCR reaction and WGS[6] (Figure 1E).

**PRACTICAL APPLICATION**

Phenotypes of organisms are fundamentally encoded within their genomes. Synthetic genomics is a cutting‐edge technology that aims to redesign natural genome sequences and de novo construct new genomes. With synthetic genomes, we are able to better understand gene functions, genome evolutions and genotype‐phenotype relationships. However, it is laborious to synthesize a genome since genomic‐scale DNA molecules are large and sequences are complex. Therefore, genomic markers are necessary to identify and quantify synthetic genomes efficiently by distinguishing specific sequences. This review summarizes the types, design approaches and applications of genomic markers utilized in synthetic genomes, which provides new insights into reprogramming organisms with targeted functions, constructions of cellular factories, and DNA data storage.

Similarly, thousands of short recoded sequences are designed within ORFs of synthetic yeast chromosomes, called PCRTags [8]. Every PCRTag is genome‐wide unique and can be used as a PCR primer that is specific to either the wild‐type or synthetic version of that ORF. Without altering corresponding peptide sequences, PCRTags serve as closely spaced genomic markers for verifying the introduction of synthetic sequence and the removal of native sequence by PCR reactions. For example, 339 distinguishable PCRTags were designed in synthetic yeast chromosome V (synV) [9]. Among these PCRTags, a series of DNA sequences in *YER172C* gene were synonymously recoded from wild‐type PCRTag sequence “TTT AAA GCT CAC CGA ACC AGA AGA GGT GTG” (WT‐PCRTag) to synthetic PCRTag sequence “CTT GAA TGA AAC GCT GCC GCT GCT AGT ATG” (SYN‐PCRTag) (Figure 1F). The synthetic PCRTag amplicon was exclusively produced when SYN‐PCRTag was used as a primer, revealing the presence of synV sequences and absence of native sequences.

Besides, sense codons are genome‐wide replaced by synonymous substitutions in both the synthetic *E. coli* genome and *S. cerevisiae* chromosomes, including stop codon TAG and serine codons TCG and TGA (Figure 1G) [7–13, 30]. The sequence of synthetic *E. coli* Syn61 was designed in silico, in which stop codons TAG were recoded to TAA, serine codons TCG were recoded to AGC, and serine codons TCA were recoded to AGT [30]. All alterations were introduced into Syn61 by genome synthesis. Overall, 18,218 target codons were coded to their target synonyms, generating a codon‐recoded *E. coli* genome with 61 codons from 64 codons. Similarly, TAG stop codons are swapped to TAA in synthetic yeast chromosomes. For example, the verified gene *YDL017W* and dubious gene *YDL016C* are overlapping in synIV [29]. In this case, stop codon TAG of *YDL017W* was recoded to TAA which did not alter the function of *YDL016C*. Recoded codons serve as closely spaced genomic markers for verifying the incorporation of synthetic sequences by using PCR reaction and WGS.

## 3 APPLICATIONS OF GENOMIC MARKERS

**FIGURE 2:**
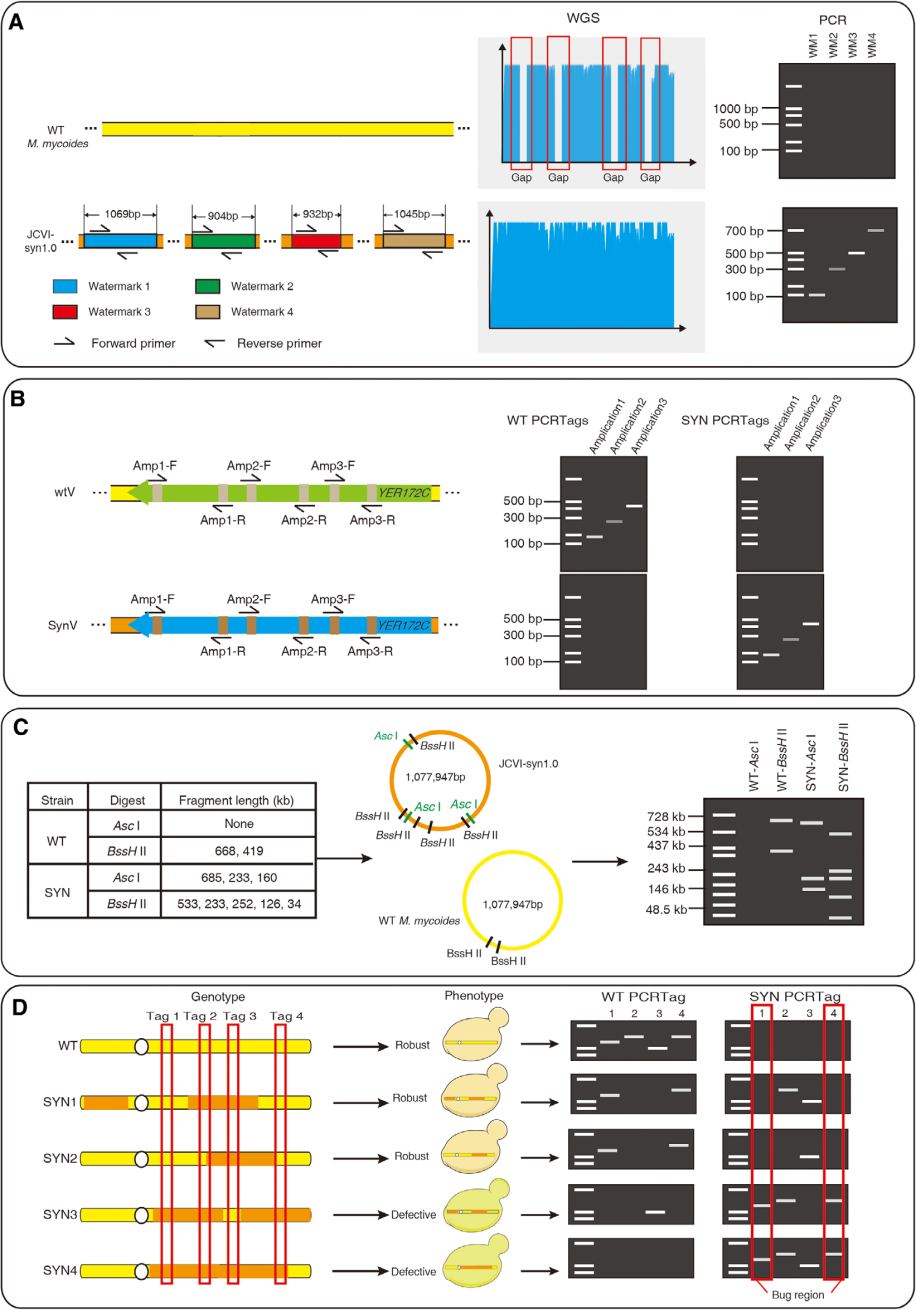
Application of genomic markers. (A) Watermarks can be employed to verify the synthetic genome by using WGS or PCR analysis. Sequencing reads covering watermarks could be only extracted from sequencing data of synthetic genome samples. Primers are designed specific to watermarks and PCR amplicons could be only detected from synthetic genomes. (B) Verification of synthetic yeast chromosomes by using PCRTags. Synthetic PCRTags are specific to synthetic genomic DNA and wild‐type PCRTags are specific to corresponding native genome. Thus, only synthetic PCRTag amplicons could be detected from the synthetic yeast chromosome. (C) Identification of the synthetic genome by restriction enzyme digestion. Restriction fragment numbers and corresponding sizes are indicated in CHEF gel. (D) Mapping defective regions on a synthetic yeast chromosome of by using PCRTags. PCRTagging analysis is employed to test the genotype of both robust and defective strains. Due to the defect should be only caused by synthetic sequences and designs, synthetic amplicons only detected in defective strains but not in robust strains were candidate bugs

Genomic markers are references for precisely representing the synthetic genome. Various methods have been employed to verify and quantify the synthetic content of genome, including WGS, PCR and enzyme digestion, etc. WGS is the gold standard for synthetic genome verification. If the native genome is successfully replaced by corresponding synthetic one, sequencing reads covering genomic markers could be extracted from the sequencing data. In contrast, these sequencing reads cannot be detected from sequencing data of wild‐type strains. Four watermarks were designed in the JCVI‐syn1.0 genome and the strain carrying a successfully assembled genome was sequenced. Watermarks were confirmed by aligning reads against the JCVI‐syn1.0 reference genome [5] (Figure 2A).

Both watermarks and short recoded sequences can be verified by using PCR‐based method. In the JCVI‐syn1.0 genome, primers were designed to specifically amplify watermarks that were confirmed by the appearance of PCR amplicons [5] (Figure 2A). In the synthetic yeast genome, PCRTags were used as PCR primers that were specific to either the synthetic genomic DNA or wild‐type one. The absence of wild‐type PCRTag amplicon and the presence of synthetic PCRTag amplicon revealed the replacement of native chromosome by synthetic one [9] (Figure 2B).

The complete assembly of a synthetic genome can be demonstrated by using restriction analyses [7, 14]. Intact genomic DNAs of both synthetic and wild‐type are first digested with specific restriction enzyme, and then are analyzed with clamped homogeneous electrical field (CHEF) gel electrophoresis. The restriction pattern of synthetic genome is distinct from that of the wild‐type one. There are two *Asc*I restriction sites on the circular JCVI‐syn1.0 genome while none is on the wild‐type genome [5]. After *Asc*I restriction enzyme digestion, the successfully assembly of JCVI‐syn1.0 was indicated by three correct restriction fragments with 685‐, 233‐, and 160‐kb in length. In contrast, no restriction fragment was detected while wild‐type *M. mycoides* genomic DNA was treated [5] (Figure 2C).

There are numerous sequence alterations on synthetic genomes, which may lead to unexpected design flaws and cause defective growth phenotypes of a synthetic strain [9, 10, 12]. The dense genomic markers, especial PCRTags, are ideal landmarks to segregate synthetic sequences and to locate defective loci. PCRTagging analysis should be carried out to analyze genotypes of both robust and defective strains [12, 31]. The synthetic amplicons only detected in defective strains but not in robust strains were candidate defective loci (Figure 2D). The yeast strain carrying initial synthetic chromosome VI (synVI) exhibited a respiratory growth defect [10]. Using this method, the defective loci was precisely narrowed down to the recoded *PRE4* gene [10]. Besides, the causes of growth defects of synthetic yeast chromosome X (synX) were pinpointed to the genomic loci including a specific synonymous recoding of the essential gene *FIP1* and the deletion of *tR(CCU)J* [12].

## 4 DISCUSSION

De novo genome synthesis advances the research of genome minimization [6], genomic recoding [7–13, 30, 32], and directed genome evolution [7, 20, 21, 25, 26], etc. The creation of these imaginative genomes is extremely challenging and laborious [3]. Thus, various genomic markers are designed to facilitate the verification and quantification of synthetic genomes, as well as the location of potential design flaws. We summarized types and applications of genomic markers that have been utilized in genome syntheses.

Genomic markers are developing from only serving as single‐functional watermarks to multifunctional elements. Inserted loxPsym sites enable synthetic yeast genomes the ability of rearrangement and evolution [16, 26], and reassignments of recoded codons on synthetic genomes enables unnatural amino acid introduction, novel polymer synthesis, viral resistance and biocontainment [33]. As large synthetic genomes of animals and plants are approaching, more diversified genomic markers are necessary to advance the precisely investigation of genome synthesis and genome evolution [34, 35]. Synthetic genomes are assembled in donor cells and subsequently transplanted to recipient cells. The epigenetic modification of genomic DNA is important to protect the synthetic genome from the restriction system of recipient cells [5]. Thus, types of genomic markers may expand from DNA level to epigenetic level such as methylation, phosphorylation to facilitate the identification of synthetic genomes.

Rational designs are required for intensively spaced genomic markers on synthetic genomes to generate specific sequences and minimized interferences with cell viability. Though aided by computer, the genomic marker design is a laborious and time‐consuming work and design flaws happen occasionally [10, 12]. Methodologies were recently developed to quantify effects of designed genomic markers for 13 genes and generated watermarks without altering gene functions [36]. With the employment of artificial intelligence (AI), deep learning and neural network, more reliable and more efficient genomic markers are capable to be generated to accelerate the synthesis, identification and characterization of synthetic genomes. Furthermore, automated biofoundries should be employed to facilitate the application of genomic markers and investigation, promoting the understanding of phenotypic impacts of genomic structural variations [37, 38, 39, 40, 41].

## CONFLICT OF INTEREST

The authors have declared no conflict of interest.

## Data Availability

Data sharing not applicable to this article as no datasets were generated or analyzed during the current study.
